# Amiodarone treatment in cats: evaluation of indications, adverse effects, and survival outcomes

**DOI:** 10.3389/fvets.2024.1509425

**Published:** 2025-01-17

**Authors:** Graham C. Rossi, Sonja S. Tjostheim, Heidi B. Kellihan, Rebecca L. Stepien, Michael Liou, Cecilia Marshall, Kathy N. Wright

**Affiliations:** ^1^Department of Medical Sciences, School of Veterinary Medicine, University of Wisconsin, Madison, WI, United States; ^2^Department of Statistics, School of Computer, Data and Information Sciences, University of Wisconsin, Madison, WI, United States; ^3^Veterinary Specialty Services, Manchester, MO, United States; ^4^MedVet Cincinnati, Fairfax, OH, United States

**Keywords:** feline, antiarrhythmic medication, tachyarrhythmias, cardiac, toxicity

## Abstract

**Introduction:**

Time-and dose-dependent adverse effects of amiodarone have not been described in cats. The primary aim of this retrospective multicenter cohort study was to report the type and frequency of clinical adverse effects and biochemical changes in cats receiving amiodarone chronically. The secondary aim was to report survival outcomes in this population of cats.

**Methods:**

Medical records were reviewed for signalment, arrhythmia diagnosis, presence of structural heart disease, systemic comorbidities and congestive heart failure at presentation, amiodarone dose, serial bloodwork results, adverse events, and survival outcome.

**Results:**

The study population included 27 client-owned cats (2016–2022). All cats had structural cardiac disease, and many were in congestive heart failure (17/27; 63%) at presentation. Amiodarone was most commonly prescribed for ventricular tachycardia (19/27, 70%), and it was administered once daily with a median [range] dose of 8.8 [4.515.2] mg/kg/day. There was a decrease in serum concentration of alanine transaminase between pretreatment values and values measured during the early amiodarone treatment window, 1–90 days (*n* = 16; *p* = 0.034). No statistical difference in serum concentration of alanine transaminase (*n* = 10; *p* = 0.799) was noted after 90 days of treatment compared to pretreatment. There was no change in hematocrit, neutrophil count, and serum concentration of alkaline phosphatase and total thyroxine during treatment in assessed cats. Ten cats (37%) had at least one episode of hyporexia or vomiting while receiving amiodarone. The median survival time for all-cause mortality was 441 days (95% confidence interval, 126–929 days); cats in which the primary therapeutic target was both supraventricular and concomitant ventricular tachyarrhythmias had at least a two-fold risk of dying compared to cats with supraventricular tachyarrhythmias alone (hazard ratio 12.9, 95% CI 1.86–89.8; *p* = 0.010).

**Discussion:**

Amiodarone was primarily used to treat ventricular arrhythmias. Transient gastrointestinal signs were reported in approximately one-third of the cats studied, but no clinically significant laboratory abnormalities were found in cats receiving amiodarone.

## 1 Introduction

Amiodarone is a broad-spectrum antiarrhythmic medication used in humans and animals for management of supraventricular and ventricular arrhythmias (1–10). It is predominantly known for its class III antiarrhythmic effect: potassium channel blockade (IK_r_ and IK_s_) resulting in prolongation of the effective refractory period which increases the action potential duration in cardiac myocytes (11). In addition, amiodarone exhibits strong sodium blockade by binding inactivated sodium channels, as well as ancillary L-type calcium channel inhibition and non-competitive beta-receptor antagonism, all of which contribute to slowed conduction. These multimodal effects support its use in therapy of both supraventricular tachycardias (SVT) and refractory ventricular tachycardias (VT) (1–3).

Time- and dose-dependent adverse effects associated with acute and chronic amiodarone administration have been documented in people and in dogs. Reported adverse effects in people include gastrointestinal signs, liver toxicity, thyroid dysfunction, keratopathy, cytopenias, peripheral neuropathy, pulmonary fibrosis, skin discoloration, and QT prolongation predisposing to torsades de pointes and increased risk of sudden death (12–19). Dogs treated with amiodarone have been reported to develop primarily gastrointestinal distress, while other complications reported in humans, such as liver toxicity, thyroid dysfunction, keratopathy, and cytopenia, appear to be uncommon in this species (4–7, 20–23). The neurological, pulmonary, and cutaneous adverse effects described in people have not been reported in dogs, suggesting that there may be species differences in the adverse effect profile of amiodarone.

The current literature on the use of amiodarone in cats is limited to two case reports documenting its use in cats affected by VT that were refractory to other antiarrhythmic therapies (9, 24), as well as an experimental study on healthy cats receiving both standard canine doses (8–10 mg/kg q24h) and high canine doses (15 and 25 mg/kg q24h) of amiodarone (10). Interestingly, this study found that both dose ranges of amiodarone were generally well-tolerated, with only some electrocardiographic changes observed, including decreases in heart rate and prolongation of the QT interval noted at all dosages and prolongation of PR interval and P wave duration noted only at higher dosages (10). No studies to date have reported the evaluated short-term or long-term clinical or biochemical tolerance of amiodarone use in a population of cats with tachyarrhythmias due to naturally occurring structural cardiac diseases.

The primary aim of this study was to assess the frequency, type, and timing of adverse clinical effects and biochemical changes in cats receiving amiodarone for the chronic treatment of naturally acquired tachyarrhythmias. The secondary aim was to report survival outcomes in this population of cats. We hypothesized that chronic amiodarone administration would be associated with gastrointestinal upset, liver enzyme elevation, thyroid dysfunction, and blood dyscrasias.

## 2 Materials and methods

Medical records of cats that received amiodarone between January 2016 and August 2022 at UW Veterinary Care (UWVC) at the University of Wisconsin, MedVet Cincinnati (MVC) and Veterinary Specialty Services (VSS) in Manchester, MO were reviewed and included in data analysis. Data were included for analysis if patients were discharged from the hospital receiving oral amiodarone (amiodarone hydrochloride, Advagen Pharma Limited, Plainsboro, NJ) at any dose and had at least one follow-up evaluation. Cats initially treated with parenteral amiodarone (Nexterone, Baxter Healthcare, Deerfield, IL) were included if the patient was then transitioned to an oral formulation. Cats receiving other antiarrhythmic medications as well as cats with cardiac and non-cardiac comorbidities were included. All cats had a complete physical examination performed by a diplomate or a resident under the direct supervision of a diplomate in the American College of Veterinary Internal Medicine specialty of cardiology. Echocardiographic and electrocardiographic examinations performed by a cardiology diplomate or a cardiology resident under the direct supervision of a diplomate. Electrocardiographic diagnoses were confirmed by the authors (GCR, KNW, CM) using available electrocardiograms (ECG), telemetry, and implantable loop recordings. No Holter assessments were performed in this cohort of patients.

Signalment, physical examination findings, ECG findings, radiographic findings, echocardiographic diagnosis, presence of congestive heart failure (CHF), medications, bloodwork results, and comorbidities were recorded. Indications for amiodarone treatment were categorized as the primary therapeutic target of SVT, VT, or both SVT and VT. Congestive heart failure was defined as the presence of pulmonary edema, pleural effusion, pericardial effusion, or a combination of two or more of these findings, in cats with atrial enlargement.

Laboratory test results were reviewed, and hematocrit (HCT), neutrophil count, platelet count, and serum concentrations of alanine transaminase (ALT), alkaline phosphatase (ALP), and total thyroxine (TT4) were recorded and grouped by timeframe. Pretreatment laboratory values were defined as the most recent bloodwork results up to 60 days prior to starting amiodarone. Posttreatment laboratory values were grouped as 1–90 days or >90 days after initiation of amiodarone therapy. If a cat had multiple laboratory values within a single timeframe, the mean lab value was used as representative of the timeframe. Incidents of hyporexia or anorexia, vomiting, diarrhea, and corneal abnormalities during treatment with amiodarone were recorded. Any abnormal gastrointestinal event was scrutinized to determine if amiodarone administration was suspected to have caused the gastrointestinal signs based on timing (< 7 days after starting amiodarone) or clinical response to amiodarone dose reduction.

Endpoints were defined as death, euthanasia, or discontinuation of amiodarone treatment. When possible, the reason for death or euthanasia was further categorized as cardiac or non-cardiac. Cardiac death was defined as sudden death, euthanasia related to the acute onset of aortic thromboembolism, or CHF signs resulting in euthanasia or death. Sudden death was defined as either witnessed death or discovery of a deceased animal that had experienced no premonitory signs of illness within the past 24 h. When imaging was not performed, acute CHF was defined as signs of tachypnea or dyspnea immediately prior to death. Referring veterinarians, clients, or both were contacted when information was missing from the medical record.

### 2.1 Statistical analysis

Data analysis was carried out in GraphPad Prism (version 9.4.1 for Windows, GraphPad Software Inc, San Diego, California; USA) and R (version 4.4.0, R Foundation for Statistical Computing, Vienna; Austria). All variables were analyzed using nonparametric methods due to the small population size. Nonparametric methods are used in location testing for robustness to non-normally distributed data. Descriptive statistics are reported as median and range, while laboratory values are reported as median and interquartile range. Percentages were rounded to the nearest whole number. Significance levels are set to α = 0.05 throughout all analyses.

Wilcoxon signed rank tests were used to compare pretreatment laboratory values with laboratory values that fell within recheck timeframes of 1–90 days or > 90 days after starting amiodarone. Confidence intervals around the (pseudo-)median are constructed by the Bauer method (25), in which we strive for 95% coverage unless limited by small sample size where noted. Longitudinal analyses were performed to identify trends in [ALT] and [ALP] over time. All laboratory values were modeled separately as a lognormal distribution for analysis, with model fit reviewed graphically. The log-linear mixed-effects model had fixed pretreatment covariates of initial ECG diagnosis (SVT vs. VT vs. SVT and VT) and lab values, presence of CHF at presentation (yes vs. no), and a random effect for individual subject. Time was modeled relative to first prescription of amiodarone. Varying slopes across time for pretreatment covariates were considered for each laboratory value. Bayesian information criteria was used for identifying model improvements and the likelihood ratio test was used for specific model comparisons. Hypothesis tests in longitudinal models are done for slope of laboratory values in the lognormal regression and testing degrees of freedom for the denominator for slopes use the Kenward–Roger approximation.

Endpoint analyses are presented with Kaplan–Meier curves. Cats were right-censored in the analysis if they were still alive at the end of the study period, if they were lost to follow-up, or if amiodarone had been discontinued during the study period. We report the log-log transformed confidence interval for median survival time. A Cox proportional hazards model was used to investigate the effect of initial ECG diagnosis and presence of CHF on overall survival.

## 3 Results

Thirty cats received amiodarone during the study period between 2016 and 2022. Eleven of 30 cats (37%) were treated at UWVC, 7/30 cats (23%) were treated at MVC, and 12/30 cats (40%) were treated at VSS. Three cats from UWVC were excluded from analysis; two did not survive to discharge, and one cat received parenteral amiodarone in hospital but was not treated with oral amiodarone due to minimal improvement in VT. The remaining 27 cats had a median age of 9.0 years (range: 0.5–16.1 years) and median body weight of 4.9 kg (range: 3.3–7.8 kg). The majority (23/27; 85%) were male. Structural cardiac disease was identified in all cats and included hypertrophic cardiomyopathy phenotype (11/27; 41%), dilated cardiomyopathy phenotype (6/27; 22%), nonspecific cardiomyopathy phenotype (5/27; 19%), restrictive cardiomyopathy (2/27; 7%), arrhythmogenic right ventricular cardiomyopathy (1/27; 4%), mitral valve dysplasia (1/27; 4%), and mitral valve dysplasia with nonspecific cardiomyopathy phenotype (1/27; 4%). Congestive heart failure was present in 17/27 cats (63%) at the time that amiodarone was prescribed. One or more comorbidities were reported in 9/27 cats (33%) at initiation of amiodarone and included azotemia (4/9; 44%), hypokalemia (3/9; 33%), aortic thromboembolism (1/9; 11%), moderate pulmonary hypertension (1/9; 11%), hyperglobulinemia (1/9; 11%), anemia (1/9; 11%), feline leukemia virus and feline immunodeficiency virus infection (1/9; 11%), cervical and pulmonary masses (1/9; 11%), lower airway disease (1/9; 11%), geriatric-onset seizures (1/9; 11%), and inflammatory bowel disease (1/9; 11%).

### 3.1 Indications and dosing

An in-clinic ECG (6-lead ECG or 6-lead ECG and continuous telemetry monitoring) was performed in all 27 cats; 2/27 cats also had an implantable loop recorder, though only one was placed before amiodarone initiation. The primary therapeutic target for amiodarone treatment was identified using the in-clinic ECG in 26/27 cats while the implantable loop ECG recording was used in 1/27 cats. The primary therapeutic target for which amiodarone was prescribed was SVT in 8/27 cats (30%), VT in 16/27 (59%), or a combination of SVT and VT in 3/27 cats (11%; Supplementary Table 1). Of the cats with SVT, 4/11 (36%) had atrial fibrillation, 3/11 (27%) had focal atrial tachycardia, 1/11 (9%) had orthodromic atrioventricular reciprocating tachycardia, 1/11 (9%) had a narrow complex tachycardia of indeterminant origin, and 1/11 (9%) had paroxysmal atrial fibrillation as well as a wide complex tachycardia of which the differential diagnoses included pre-excited atrial fibrillation (previously documented ventricular pre-excitation), or VT. Orthodromic atrioventricular reciprocating tachycardia was suspected but not confirmed in one cat (1/11, 9%) with ventricular pre-excitation and repeated events of weakness, lethargy, hypersalivation, and open-mouth breathing.

Six cats (*n* = 3, UWVC; *n* = 3, MVC) received intravenous amiodarone prior to oral therapy, administered as a 2–5 mg/kg bolus over 10–15 min, followed by 0.4–0.6 mg/kg/h continuous rate infusion for 6–8 h and then 0.2–0.3 mg/kg/h for 6–18 h. At UWVC, oral amiodarone therapy began with either a 7-day (*n* = 4) or 14-day (*n* = 4) loading protocol. For the 7-day loading protocol, the median dose was 12.7 mg/kg/day (range: 9.8–15.6 mg/kg/day). Amiodarone was administered every 24 h during the 7-day loading protocol in all 4 cats. The 14-day loading protocol involved a higher median dose during the first week, 29.7 mg/kg/day (range: 9.1–31.1 mg/kg/day), followed by a lower median dose during the second week, 14.9 mg/kg/day (range: 9.1–15.6 mg/kg/day). During the 14-day loading protocol, amiodarone was administered every 12 h in three cats and every 24 h in one cat. Both protocols transitioned to a maintenance dose, with a median dose of 7.2 mg/kg/day (range: 4.5–7.8 mg/kg/day). Cats treated at MVC and VSS did not receive oral loading doses; maintenance therapy was initiated at the time of arrhythmia diagnosis with a median dose of 10.2 mg/kg/day (range: 6.4–15.2 mg/kg/day). The median maintenance dose for the entire population was 8.8 mg/kg/day (range: 4.6–15.2 mg/kg/day), with all cats receiving amiodarone every 24 h. The owner of one cat increased the amiodarone dosing from once daily to every 12 h after 11 weeks for perceived tachycardia based on chest palpation. In another cat, the amiodarone dose was decreased from 30 mg administered every 24 h (7.1 mg/kg/day) to 20 mg administered every 24 h (6.7 mg/kg/day) after 5 months by the prescribing clinician following the patient's significant weight loss.

### 3.2 Other medications

Most cats (16/27; 59%) were treated with at least one other antiarrhythmic medication prior to starting amiodarone. In 9/16 cats (56%) the other antiarrhythmic agents [sotalol, 6/9 (67%); diltiazem, 1/9 (11%); atenolol, 2/9 (22%); lidocaine, 2/9 (22%); procainamide, 2/9 (22%); magnesium sulfate, 1/9 (11%)] were discontinued before or at the time that amiodarone treatment was started. Seven of 16 cats (44%) were discharged from hospital receiving the combination of amiodarone and at least one other antiarrhythmic agent: sotalol (3/7; 43%), diltiazem (2/7; 29%), atenolol (1/7; 14%), and sotalol plus diltiazem (1/7; 14%). Six cats continued to receive the other antiarrhythmic drug long-term; the sotalol dose was tapered over 7 days after discharge and then discontinued in one cat. Information about non-antiarrhythmic medications given at the time of amiodarone initiation was only available from one institution (8/27; 30%); most were related to therapy of CHF, including pimobendan (6/8; 75%), furosemide (5/8; 63%), enalapril (1/8; 13%), spironolactone (1/8; 13%), clopidogrel (6/8; 75%), dalteparin (1/8; 13%), potassium chloride (2/8; 25%), mirtazapine (2/8; 25%), and maropitant (1/8; 13%).

### 3.3. Adverse effects

Gastrointestinal signs were reported at presentation prior to treatment with amiodarone in 11/27 cats (41%) and included hyporexia alone (7/11; 64%), hyporexia with vomiting (2/11; 18%), vomiting alone (1/11; 9%), or diarrhea alone (1/11; 9%). These signs resolved without therapy and were not reported again in seven of these cats; four cats had recurrence of hyporexia, vomiting, or both during the study period. After starting amiodarone treatment, gastrointestinal signs were reported in 10/27 cats (37%) and included hyporexia alone (8/10; 80%) or hyporexia with vomiting (2/10; 20%). Diarrhea was not reported in any cat during the study period. Amiodarone is suspected to have contributed to the development of gastrointestinal signs in 4/10 cats (hyporexia alone *n* = 2, hyporexia with vomiting *n* = 2) based on timing of newly reported gastrointestinal sign (within 7 days, *n* = 3) or because of an apparent response to amiodarone dose reduction (*n* = 1). Two cats (2/4; 50%) concurrently received first-time or escalated therapy for CHF and had new or progressive azotemia; amiodarone was discontinued in one of these cats due to persistent hyporexia following 3 days of administration. Amiodarone may have contributed to late onset hyporexia, reported in 6/10 cats (60%) after 1–14 months of receiving the drug. At the time the cause of the hyporexia were attributed to systemic illness in 4/6 cats [recurrent upper respiratory infection (*n* = 1), severe periodontal disease (*n* = 1), gastrointestinal bleeding attributed to dalteparin administration (n = 1), and presumptive flare-up of historical inflammatory bowel disease (*n* = 1)], though possible additive effects of chronic amiodarone therapy in conjunction with systemic illness may have contributed to these cats' clinical gastrointestinal signs.

Most cats underwent some type of pretreatment laboratory testing. Specifically, 23 cats had a chemistry profile with a median time of 0 days (range: −42 to 0 days) before the start of oral amiodarone. Thirteen cats had a complete blood cell count (CBC) with a median time of 1 day (range: −42 to 0 days) before the start of oral amiodarone. Seven cats had a measurement of TT4 with a median time of 0 days (range: −10 to 0 days) before the start of oral amiodarone. Comparisons of pretreatment laboratory values to values performed 1–90 days or >90 days after starting amiodarone are displayed in Tables 1, 2.

**Table 1:** Comparison of laboratory values before (pretreatment) and after (1–90 days) initiation of amiodarone therapy.

**Laboratory parameter**	**Pretreatment**	**1–90 days**	**Treatment days**	***p*-value**	**Difference CI**	**CI level**
ALT (U/L)	69 (44–139) *n* = 23	47 (37–93) *n* = 19	23 [3–89]	0.034^*^	−12.1 (−132.5, −0.5)	95%
	# paired lab values = 16				
ALP (U/L)	33 (20–54) *n* = 23	40 (36–59) *n* = 20	23 [3–89]	0.737	−1 (−9.0,−6.5)	95%
	# paired lab values = 17				
HCT (%)	44 (38–47) *n* = 13	38 (37–40) *n* = 7	29 [8–65]	0.197	−8.1 (−16.5, 0)	80%
	# paired lab values = 4				
Neutrophils (k/μL)	7.8 (5.7–11.9) *n* = 13	5.1 (4.0–7.9) *n* = 8	27 [7–65]	0.080	−2.2 (−3.6,−0.3)	90%
	# paired lab values = 5				
Total T4 (μg/dL)	2.5 (1.4–2.8) *n* = 7	2.5 (2.3–2.7) *n* = 8	33 [13–61]	0.285	−0.44 (−0.2, 1.0)	80%
	# paired lab values = 3				

The laboratory values are presented as median (interquartile range), and total number of cats tested within each timeframe (n). The number of treatment days at the time of the recheck exam are presented as median [range]). Difference confidence intervals (CI) are around (pseudo-)median of the paired differences, not difference of medians. Confidence level goal is 95% but are not attained for all laboratory values due to small sample size.

ALT, alanine aminotransferase; ALP, alkaline phosphatase; CI, confidence interval; HCT, hematocrit; T4, thyroxine.

^*^Denotes statistical significance.

**Table 2:** Comparison of laboratory values before (pretreatment) and after (>90 days) initiation of amiodarone therapy.

**Laboratory parameter**	**Pretreatment**	**>90 days**	**Treatment days**	***p*-value**	**Difference CI**	**CI level**
ALT (U/L)	69 (44–139) *n* = 23	70 (55–112) *n* = 13	226 [100–695]	0.799	3.3 (−65.0, 24.2)	95%
	# paired lab values = 10				
ALP (U/L)	33 (20–54) *n* = 23	49 (29–55) *n* = 14	241 [100–695]	0.859	1.3 (−16.4, 14.0)	95%
	# paired lab values = 11				
HCT (%)	44 (38–47) *n* = 13	40 (38–48) *n* = 6	302 [100–462]	0.144	−7.5 (−12.5, −1.7)	80%
	# paired lab values = 4				
Neutrophils (k/μL)	7.8 (5.7–11.9) *n* = 13	5.3 (3.6–8.3) *n* = 6	302 [100–462]	0.273	−3.0 (−5.9, 0.1)	80%
	# paired lab values = 4				
Total T4 (μg/dL)	2.5 (1.4–2.8) *n* = 7	3.0 (2.3–3.7) *n* = 4	219 [140–462]			
	# paired lab values = 1				

The laboratory values are presented as median (interquartile range), and total number of cats tested within each timeframe (n). The number of treatment days at the time of the recheck exam are presented as median [range]. Difference confidence intervals (CI) are around (pseudo-)median of the paired differences, not difference of medians. Confidence level goal is 95% but are not attained for all laboratory values due to small sample size.

ALT, alanine aminotransferase; ALP, alkaline phosphatase; CI, confidence interval; HCT, hematocrit; T4, thyroxine.

**Figure 1:**
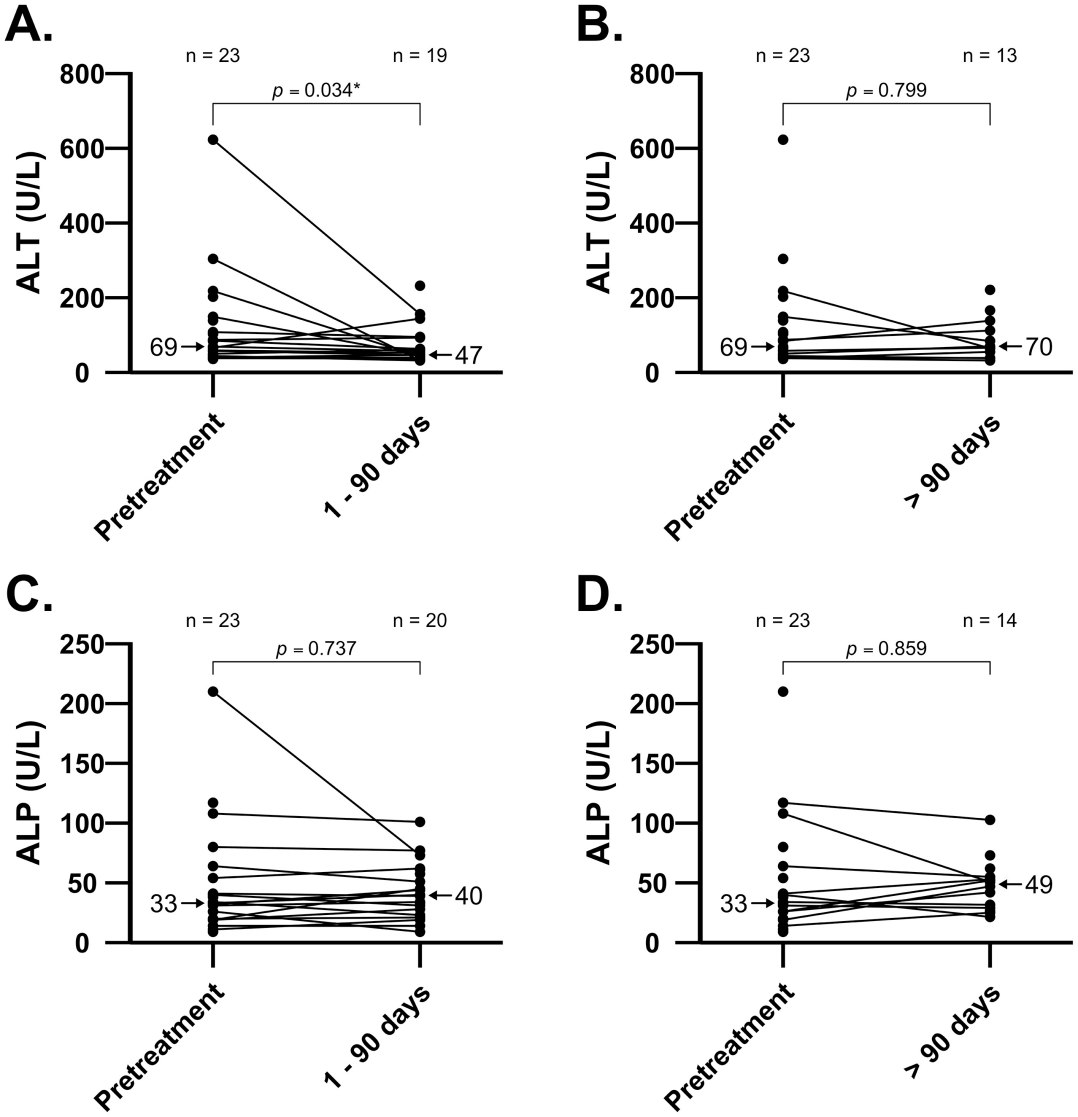
Individual values in [ALT] and [ALP] before and after short-term [0–90 days; panel **(A)** and **(C)**] and long-term [>90 days; panel **(B)** and **(D)**] treatment with amiodarone in 23 cats. Comparison of [ALT] between pretreatment and short-term treatment with amiodarone shows median decrease (*p* = 0.034). There were no increases in median [ALT] or [ALP] in cats during short-term or long-term treatment with amiodarone. Median values are indicated by arrows. ALT, alanine aminotransferase; ALP, alkaline phosphatase.

Twenty-four of 27 cats (89%) had a chemistry panel performed at least once while receiving amiodarone. Not all cats had pretreatment laboratory values for comparison; 18 cats had pretreatment [ALT] and at least one posttreatment [ALT], while 20 cats had pretreatment [ALP] and at least one posttreatment [ALP]. There was a median decrease in [ALT] (*n* = 16, *p* = 0.034; Figure 1) from pretreatment to 1–90 days, but no change in [ALT] between pretreatment and >90 days (*n* = 10, *p* = 0.799; Figure 1).

Seventeen of 23 cats (74%) had a normal pretreatment [ALT] (reference interval: 20–108 U/L). The [ALT] had increased in 6/18 cats (33%) in the first follow-up panel after initiation of amiodarone therapy; [ALT] remained within the reference interval in all but two of these cats. Of the two cats in which [ALT] was outside of the reference range, one had hyporexia and vomiting 1 week after starting amiodarone while tapering the sotalol dose. Biochemistry revealed elevated [ALT] at 114 U/L (pretreatment ALT 66 U/L; 1.7-fold increase) and concurrent mild azotemia (creatinine 1.9 μg/dL) with no other biochemical abnormalities. The amiodarone loading dose (15.6 mg/kg/day) was decreased to maintenance dosing (7.8 mg/kg/day), sotalol was discontinued, and supportive care was provided, resulting in clinical resolution of gastrointestinal signs. The cat was re-presented for open mouth breathing and an uncontrolled tachyarrhythmia 5 weeks later. At this visit, [ALT] was further elevated at 174 U/L and the cat was euthanized due to an incessant wide-complex tachyarrhythmia, thought to be ventricular tachycardia or pre-excited atrial fibrillation, and declining clinical status. The second cat had a 2.9-fold change as measured after 105 days on amiodarone and was asymptomatic. No dose adjustment was made and this cat had a normal [ALT] at the time of the next recheck, 410 days after starting amiodarone.

A minority of cats (6/23; 26%) had elevated pretreatment [ALT], with most (5/6; 83%) showing signs of CHF at that time. Two of these cats (33%) had a markedly lower [ALT] at the next evaluation (pretreatment: 623 and 292 U/L, post-treatment: 156 and 113 U/L respectively), while 3/6 cats (50%) had an [ALT] within reference range at second evaluation. The last cat in this group was euthanized 12 days after starting amiodarone, despite marked clinical improvement, and a chemistry panel was not performed.

Most cats (14/23; 61%) had a pretreatment [ALP] within the expected reference interval (23–107 U/L). The pretreatment [ALP] was above the reference range in three cats; the [ALP] was within the reference range after starting amiodarone in two of these cats, while the third cat had a mild [ALP] elevation that persisted but did not change significantly on follow up biochemistry panels. Six of 9 cats with abnormal pretreatment [ALP] rechecked in the normal reference interval.

Thirteen cats (13/27; 48%) had a CBC performed prior to starting amiodarone and 7/13 cats (54%) had at least one follow-up CBC while receiving amiodarone. Most cats (6/7; 86%) had neutrophil counts and HCT within reference range at all available posttreatment timepoints. One cat developed hyporexia 25 days after starting amiodarone and a CBC revealed a new neutropenia (1.0 k/μL, reference interval 1.5–9.6 k/μL; pretreatment 2.3 k/μL) and a new normocytic hypochromic anemia (HCT/TP: 30%/7.9 g/dL, reference interval 31–48%/6.7–8.9 g/dL; pretreatment 53%/8.5 g/dL). The hyporexia resolved with mirtazapine treatment, and the HCT and neutrophil count returned to normal without adjustment of the amiodarone dose; the hyporexia was not attributed to amiodarone administration in this patient. Automated and manual platelet counts were not consistently reported among laboratories and therefore only eight of 13 cats (62%) had a baseline CBC that also reported a platelet count. All these cats had an automated platelet count >200 k/μL or a blood smear that confirmed platelet clumping prior to treatment. Four of eight cats had at least one additional platelet count after starting amiodarone; three cats had platelet counts within reference range at recheck while one cat had a low automated platelet count [ < 200 k/μL (109–151 k/μL)] without a reported blood smear or manual platelet count.

Ten of 27 cats (37%) had [TT4] analyzed at least once while receiving amiodarone. Two of 10 cats (20%) had a mildly elevated [TT4] while receiving amiodarone based on laboratory-specific reference intervals. Neither cat had [TT4] measured prior to treatment. One cat had a [TT4] of 4.3 μg/dL (reference interval, 0.8–3.8 μg/dL) at a 6-week recheck. Treatment for hyperthyroidism was not initiated and the [TT4] was within reference range at recheck examinations performed five and 11 months later. The second cat had a [TT4] of 5.3 μg/dL (reference interval, 0.8–4.7 μg/dL) after 15 months and was then lost to follow up. No cat had [TT4] below reference intervals at any timepoint. Thyroid-stimulating hormone (TSH) and triiodothyronine (T3) concentrations were not measured in this population. Corneal changes were not reported in any cat.

Longitudinal modeling was used to quantify strength of loglinear trends over time in select laboratory values after starting amiodarone. The model did not document any statistically significant trends for [ALT] and [ALP]. There were not enough data points for HCT, neutrophil count, or [TT4] to perform meaningful longitudinal modeling.

### 3.4 Survival outcomes

**Figure 2:**
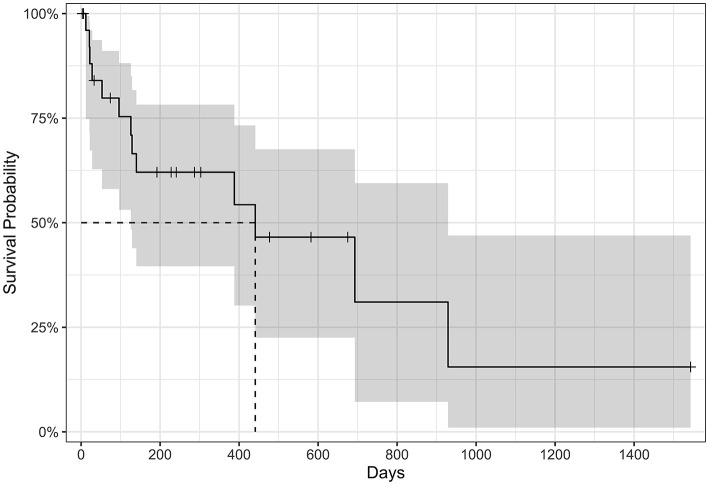
Kaplan–Meier survival curve of all-cause mortality in cats treated with amiodarone (*n* = 27). Tick marks represent censored data for cats still alive at the study end, cats lost to follow-up, or cats where amiodarone had been discontinued during the study period. The median survival time is 441 days (95% confidence interval 126–929 days noted in gray).

The MST for all-cause mortality was 441 days (95% confidence interval, 126–929 days; Figure 2). Eight of 27 cats (30%) were still alive by the end of the study period and had received amiodarone for a median of 390 days (range: 192–1,543 days). Amiodarone was discontinued in a minority of cats (3/27; 11%) due to suspected resolution of VT (based on implantable loop recorder ECGs) after 73 days (*n* = 1), no improvement in SVT after 287 days warranting a different antiarrhythmic agent (*n* = 1), or persistent hyporexia during the first 3 days of receiving amiodarone (*n* = 1). Thirteen of 27 cats (48%) were euthanized or had died by the end of data collection. Euthanasia was performed because of recurrent CHF in 2/13 cats (15%), non-cardiac causes in 4/13 cats (31%), and unknown reasons in 3/13 cats (23%). The reasons for non-cardiac death include anorexia (*n* = 2), kidney failure and suspected brain tumor (*n* = 1), and elective euthanasia due to owner fear for future deterioration and poor quality of life (*n* = 1). Four of 13 cats (31%) died at home and the cause of death is unknown. Three of 27 cats (11%) were lost to follow-up.

The initial ECG diagnosis was found to have a strong effect on survival. Cats in which the primary therapeutic target was both SVT and VT had at least a two-fold risk of dying compared to cats with SVT alone (hazard ratio 12.9, 95% CI 1.86–89.8; *p* = 0.010). This effect remains statistically significant regardless of whether the patient was in CHF or not. There is no statistical difference in survival in cats with a primary therapeutic target of VT compared to those with SVT alone (hazard ratio 2.42, 95% CI 0.48–12.2; *p* = 0.284), nor in cats with CHF compared to those not in CHF (hazard ratio 1.88, 95% CI 0.54–6.56; *p* = 0.325).

## 4 Discussion

This study reports type and frequency of adverse clinical effects and biochemical changes that occurred in cats that received chronic amiodarone for therapy of arrhythmias. Acute and chronic amiodarone therapy was tolerated in most cats, though hyporexia, and less frequently vomiting, occurred in around one third of cats (*n* = 10). These signs may have been secondary to many factors, including amiodarone, other cardiac medications, CHF, azotemia, uncontrolled arrhythmias, concurrent non-cardiac illnesses, or a combination thereof, and resulted in drug discontinuation in one cat. Pathophysiology of gastrointestinal signs associated with amiodarone therapy in people, when not directly attributed to hepatotoxicosis or acute pancreatitis, is not well described (12). In people and in dogs, clinical improvement in gastrointestinal signs is often reported following amiodarone dose reduction (6, 7, 12, 21). Gastrointestinal signs resolved following a dose reduction of amiodarone in one cat in this study. In contrast, in the single cat in which amiodarone was discontinued, hyporexia did not improve following amiodarone discontinuation and the hyporexia was suspected to be multifactorial.

Human amiodarone-associated hepatotoxicosis is uncommonly reported (1%−3% of patients), but liver enzyme elevations are reported in 15%−50% of patients receiving chronic amiodarone therapy (26). Amiodarone is presumed to cause direct damage to the hepatocyte lipid bilayer and may alter lysosomal and mitochondrial function (2). Histopathologic findings of suspected human amiodarone hepatotoxicity included steatosis, ballooning of hepatocytes, Mallory bodies, and fibrosis (27). Variable dose-dependent liver enzyme elevation and hepatotoxicosis have been associated with amiodarone treatment in dogs in some (5–7, 20, 21), but not all studies (4). In this study, two of 14 cats (14%) with normal pretreatment [ALT] later developed mild [ALT] elevation while receiving amiodarone. Only one of these cats had concurrent hyporexia and vomiting, which resolved following a dose reduction and supportive care. These results suggest that short-term and long-term amiodarone therapy does not result in persistent liver enzyme elevation and hepatoxicity in the majority of cats. Possible risk factors for amiodarone-induced hepatoxicity, such as amiodarone dose, breed variability, and effects of concurrent disease conditions or medications cannot be evaluated in this small, retrospective study, and individual surveillance is still recommended. The improvement in [ALT] that was noted at 1–90 days in five cats presented with CHF may be attributable to improved liver perfusion following initiation of CHF therapy or to improved liver perfusion after improvement in cardiac rhythm status (28–31). However, it should be noted that clinical response to therapy for heart failure and antiarrhythmic efficacy of amiodarone were not analyzed in this study. A similar trend was observed in the longitudinal modeling of [ALT] and [ALP] over time, though these results did not reach statistical significance. Cytopenias and bone marrow granulomas associated with amiodarone administration have been sporadically reported in people and gradually resolve following drug discontinuation (14, 16, 17). Pathogenesis of amiodarone-associated granuloma formation is unclear but may be related to increased tissue iodine concentrations or accumulation of a phospholipid-like substance from amiodarone-mediated inhibition of phospholipases (32). In a small number of dogs, anemia, thrombocytopenia, and neutropenias have been associated with amiodarone administration and the cytopenias were reversible following drug discontinuation or dose reduction (7, 21, 23). In the present study, a progressive absolute neutropenia and new mild anemia were documented 2 days after starting amiodarone in one cat; this cat was neutropenic prior to starting amiodarone and HCT was normal 10 months later with no change to the amiodarone dose, suggesting that this patient's bicytopenia may have been multifactorial or unrelated to amiodarone administration. There was no instance of absolute neutropenia or anemia in the remaining 26 cats reported here.

No cats in this study developed a low [TT4], and one cat had what appeared to be a transient hyperthyroidism which resolved without treatment or discontinuation of amiodarone. Amiodarone-induced thyroid dysfunction is estimated to occur in 15%−20% of human patients and includes amiodarone-induced hypothyroidism and amiodarone-induced thyrotoxicosis (33–36); in contrast, in dogs, amiodarone-induced thyroid dysfunction seems rare (4, 7). Either may result from the intrinsic drug effects of amiodarone on tissue or increased iodine intake from the high iodine concentration in amiodarone tablets. Amiodarone-induced hypothyroidism may result from inhibition of thyroxine 5′-deiodinase by amiodarone and decreased conversion of tetraiodothyronine (T4) to triiodothyronine (T3) or failure to escape from acute Wolff-Chaikoff effect (a temporary physiological phenomenon) which may be promoted by concurrent thyroid autoimmunity (33, 34). In cases where there is impaired T4 to T3 conversion, a normal [TT4] does not rule out amiodarone-induced hypothyroidism, and a more comprehensive thyroid panel that includes measurement of [T3] and [TSH] is necessary to identify impairment. A comprehensive thyroid profile was not performed in any of the cats in this study and therefore amiodarone-induced hypothyroidism may have gone undetected. Amiodarone-induced thyrotoxicosis is thought occur by two possible mechanisms: (1) increased serum iodide load resulting in enhanced thyroid hormone synthesis or (2) direct damage to thyroid follicular cells resulting in thyroiditis and release of preformed T4 and T3 into circulation (35). Cats have a high prevalence of thyroid disease, but whether feline hyperthyroidism influences susceptibility to amiodarone-induced thyroid dysregulation is unknown. The [TT4] was elevated in two cats while receiving amiodarone, but pretreatment [TT4] values were not available; these cats may have had elevated [TT4] prior to treatment. In one of these two cats, the transiently elevated [TT4] may have been related to amiodarone administration (mild amiodarone-induced thyrotoxicosis in humans is reported to resolve spontaneously in ~20% of cases) but a spurious or diet-related etiology could not be excluded (36). The second cat was 13 years old, an age where naturally occurring hyperthyroidism is overrepresented.

Corneal lesions were not reported in this population of cats, though full ophthalmic exams may not have been performed by the attending cardiologist. Amiodarone-induced corneal deposits are a common adverse effect in people (70%−100% of patients) and have been characterized by the presence of vortex-like lipid deposits of the anterior cornea containing lysosome-like intracytoplasmic inclusions and producing no loss of vision (15, 37). Bicer et al. (22) reported trace corneal deposits in one of six Beagle dogs (16%) receiving amiodarone, a considerably lower frequency compared to humans.

The variability in treatment protocols at the three hospitals in this study reflect the uncertainty that remains regarding the most appropriate initial dosing regimen for amiodarone in cats. Variability in chosen dose for acute antiarrhythmic therapy may be due to perceived risk of sudden death, clinician experience or preference, presence of CHF or unidentified causes, and the effect of this variability in dosing is difficult to discern in the setting of rapidly evolving therapeutic regimens in unstable patients. Regardless of the diagnosed arrhythmia or initial therapy, cats in this study received oral amiodarone at a median maintenance dose of 8.8 mg/kg/day, providing some degree of comparability of the chronic phase of therapy. This dose falls within the range of median maintenance doses reported in dogs with naturally occurring cardiac disease 4.3–12.1 mg/kg/day; (4, 5, 7, 21) and appears to be higher than in people, where typical maintenance doses range 100–400 mg/day (roughly 1.0–7.0 mg/kg/day for 55–90 kg adults) (2).

In humans, there is an increasing risk of experiencing an adverse drug effect with continued exposure to amiodarone, even in patients receiving a low dose treatment protocol (38). In veterinary medicine, time-dependent increases in adverse events may contribute to mortality, particularly if there is an associated perceived decrease in quality of life. Most of the cats in this report were in CHF and experiencing life-threatening arrhythmias when amiodarone therapy was initiated, and approximately a third of the population had dilated cardiomyopathy or restrictive cardiomyopathy which confer a more guarded long-term prognosis (39, 40). Despite this, the MST for all-cause mortality (441 days) was comparable to previous reports of cats with hypertrophic cardiomyopathy and CHF [MST 194–563 days (41–43)]. While this study was not designed to test for a survival benefit of amiodarone treatment, this data suggests that it did not obviously contribute to worsening survival in this population of cats.

Cats were found to have a significantly higher risk of death within this population if the primary therapeutic target was a combination of VT and SVT. There are many factors that may have contributed to this finding, including inadequate broad-spectrum arrhythmia control, more severe heart disease, compounding effects of persistent tachycardia on CHF management, poorly tolerated drug-associated adverse effects, or perceived poorer long-term prognosis. The number of cats with both VT and SVT were few and therefore a larger sample size is needed to further investigate this effect and to determine the clinical implications. The presence of CHF did not have a statistically significant impact on survivability in this cohort of cats, which contrasts with other studies (41–43). There were fewer cats not in CHF thereby lowering the power of our study which may explain the discrepant findings.

Limitations of this report are primarily related to the retrospective nature of the study. Amiodarone loading and maintenance dosing was variable between institutions. The therapeutic dose of amiodarone is not known in cats and may be higher than was reported in this study, influencing the apparent low frequency of adverse effects. Follow-up laboratory testing was not available in all cats at all timepoints, and when present, the follow-up time frame was variable. The timing of pretreatment laboratory testing was variable (in some cases, up to 60 days), limiting our ability to assess biochemical normality at the time of initiation of amiodarone therapy. The small sample size may have limited the ability to detect statistically significant differences. Hyperbilirubinemia has been reported as a rare adverse effect of amiodarone treatment in both humans and dogs (5, 19, 21). Bilirubin concentration was not evaluated in this study and therefore incidence of hyperbilirubinemia in cats remains unknown. Total thyroxine concentration was reported in a minority of cats after starting amiodarone, limiting assessment of amiodarone-induced thyroid dysfunction. Moreover, without paired TSH or T3 testing, the presence of amiodarone-induced hypothyroidism could not be evaluated in this population. Variation between hematologic and biochemical analyzers at different hospitals may influence comparability. Manual platelet count or platelet clumping were not consistently reported thus limiting interpretation of the automated platelet count. The etiology of gastrointestinal signs in this population could rarely be determined with confidence and may have been multifactorial in most cats. Screening for ophthalmologic abnormalities was not consistently documented, and therefore it is possible that corneal lesions were present but not reported. Measurements of ECG waveforms, segments, and intervals were not analyzed in this study and therefore the incidence of prolongation of PQ and QT intervals remains unknown in cats receiving amiodarone. This lack of data may have precluded not only potential analyses on the prevalence of interval prolongations associated with amiodarone administration in cats but also any investigation into the prognostic role of such electrocardiographic abnormalities, especially considering that QT interval prolongation has already been shown to influence survival in cats with left ventricular hypertrophy (44). Lastly, Holter recordings were not performed and therefore the efficacy of the antiarrhythmic or proarrhythmic effect of amiodarone cannot be accurately assessed. In cases of VT, Holter recordings can confirm the absence of VT runs after the initiation of oral amiodarone therapy, whereas a standard ECG cannot provide this certainty. Moreover, in cases of SVT, Holter monitoring is necessary to document the absence or paroxysmal relapse of SVT in cats that have been cardioverted, or to verify well-controlled mean heart rates in cats with SVT that cannot be managed with rhythm control, such as atrial fibrillation.

## 5 Conclusion

In this study, amiodarone may have contributed to transient gastrointestinal signs in approximately one-third of the enrolled cats at the reported doses, although it did not cause persistent changes in the hematologic parameters we investigated during treatment. Standardized prospective investigation of potential adverse effects, therapeutic efficacy, and clinical outcome of amiodarone use in cats with structural cardiac disease is warranted to best direct its use in this species.

## Data Availability

The original contributions presented in the study are included in the article/Supplementary material, further inquiries can be directed to the corresponding author.
