# Supplementary material for: Amiodarone treatment in cats: evaluation of indications, adverse effects, and survival outcomes
Source: Front Vet Sci. 2025 Jan 17;11:1509425. doi: 10.3389/fvets.2024.1509425 (PMC11782268; doi:10.3389/fvets.2024.1509425)
Supplement: Supplementary file 1 [file Table_1.docx]

Supplementary Material

# Supplementary Table 1: Results of the cardiology evaluation performed at the time when amiodarone treatment was started in individual cats.

| Cat | Electrocardiographic diagnosis | Primary therapeutic target | Echocardiographic diagnosis | CHF present |
| --- | --- | --- | --- | --- |
| 1 | -AF  -Multiform single VPCs | SVT | NCM | Yes |
| 2 | -Nonsustained VT | VT | NCM  MVD | No |
| 3 | -Monoform VPCs (singles, couplets, triplets) and nonsustained VT  -Periods of junctional tachycardia with isorhythmic atrioventricular dissociation (type I) | VT | NCM | No |
| 4 | -Sustained VT | VT | HCM | No |
| 5 | -OAVRT  -Single VPCs | SVT | MVD | No |
| 6 | -Sinus rhythm with ventricular pre-excitation  -Paroxysmal AF  -Wide complex tachycardia (ddx VT or pre-excited AF) | VT and SVT | HCM | No |
| 7 | -Sustained AT  -Single and couplet VPCs | SVT | NCM | No |
| 8 | -Single and couplet VPCs and nonsustained VT | VT | HCM | Yes |
| 9 | -Nonsustained AT  -Single VPCs | SVT | HCM | Yes |
| 10 | -Atrial salvoes to nonsustained runs of atrial tachycardia  -Single APCs | SVT | RCM | Yes |
| 11 | -Sinus rhythm with ventricular preexcitation  -OAVRT not documented but it was suspected as etiology for repeated events of weakness, lethargy, hypersalivation, and open-mouth breathing | SVT | DCM | No |
| 12 | -Sustained bimorphic VT | VT | HCM | No |
| 13 | -Salvoes of VT  -Single APCs | VT | HCM | Yes |
| 14 | -Sustained monomorphic VT, alternating with polymorphic VT | VT | DCM | Yes |
| 15 | -Sustained narrow complex tachycardia | SVT | DCM | Yes |
| 16 | -Salvoes of VT | VT | DCM | Yes |
| 17 | -Paroxysmal VT | VT | HCM | Yes |
| 18 | -AF  -VT followed by sinus arrest upon termination | VT and SVT | NCM | Yes |
| 19 | -Paroxysmal VT | VT | NCM | Yes |
| 20 | -Monomorphic VT | VT | HCM | Yes |
| 21 | -AF  -Salvoes of VT | VT and SVT | DCM | Yes |
| 22 | -Paroxysmal VT | VT | HCM | Yes |
| 23 | -Salvoes of VT, ventricular bigeminy | VT | ARVCM | No |
| 24 | -Paroxysmal VT | VT | RCM | No |
| 25 | -Sustained VT | VT | DCM | Yes |
| 26 | -Sustained VT | VT | HCM | Yes |
| 27 | -AF  -Single VPCs | SVT | HCM | Yes |

AF, atrial fibrillation; APCs, atrial premature complexes; ARVCM, arrhythmogenic right ventricular cardiomyopathy; AT, atrial tachycardia; DCM, dilated cardiomyopathy; HCM, hypertrophic cardiomyopathy; MVD, mitral valve dysplasia; NCM, nonspecific cardiomyopathy; RCM, restrictive cardiomyopathy; SVT, supraventricular tachycardia; VPCs, ventricular premature complexes; VT, ventricular tachycardia
